# Measles virus genotype D4 strains with non-standard length M-F non-coding region circulated during the major outbreaks of 2011-2012 in Spain

**DOI:** 10.1371/journal.pone.0199975

**Published:** 2018-07-16

**Authors:** Horacio Gil, Aurora Fernández-García, María Mar Mosquera, Judith M. Hübschen, Ana M. Castellanos, Fernando de Ory, Josefa Masa-Calles, Juan E. Echevarría

**Affiliations:** 1 National Reference Laboratory for Measles and Rubella, Centro Nacional de Microbiología, Instituto de Salud Carlos III, Majadahonda, Madrid, Spain; 2 European Program for Public Health Microbiology Training (EUPHEM), European Centre for Disease Prevention and Control (ECDC), Solna, Sweden; 3 CIBER de Epidemiología y Salud Pública (CIBERESP), Madrid, Spain; 4 WHO European Regional Reference Laboratory for Measles and Rubella, Department of Infection and Immunity, Luxembourg Institute of Health, Esch-sur-Alzette, Luxembourg; 5 Centro Nacional de Epidemiología, Instituto de Salud Carlos III, Madrid, Spain; Keele University Faculty of Natural Sciences, UNITED KINGDOM

## Abstract

In recent decades, vaccination has substantially reduced the number of measles cases to levels close to the elimination stage. However, major measles outbreaks occurred in Europe during 2010–2012, after the introduction of the D4-Enfield lineage. We have performed a molecular characterization of 75 measles virus genotype D4 strains from patients infected in Spain between 2004 and 2012 by sequencing the N-450 region and the M-F non-coding region (M-F NCR) in order to identify genetic features of these viruses. The analysis of the N-450 region confirmed that all samples obtained since 2008 belonged to variants or sets of identical sequences of the D4-Enfield lineage, including a new one named MVs/Madrid.ESP/46.10/. Analysis of the M-F NCR showed insertions and deletions associated with previously described, uncommon non-standard genome length measles viruses. This genetic feature was identified in the D4-Enfield lineage viruses, but not in the other D4 viruses that were circulating in Spain before 2008, suggesting that these non-standard length M-F NCR sequences are characteristic of the D4-Enfield lineage. The results of the phylogenetic analysis of Spanish M-F NCRs suggest higher resolution in discriminating strains than did the N-450 analysis. In addition, the results of the analysis of the M-F NCR on the MVs/Madrid.ESP/46.10/ sub-lineage seem to support the potential utility of this region as a tool for epidemiological surveillance complementary to the N-450 region, as previously suggested. Further investigation on this question, as well as the surveillance of new potentially emerging strains with non-standard length M-F NCR are strongly recommended as part of future strategies for measles elimination.

## Introduction

Measles is a highly contagious infectious disease caused by the measles virus (MeV), which continues to be a major cause of infant mortality throughout the world and of continuing outbreaks in developed countries, despite the existence of an effective live-attenuated vaccine.

In the WHO European Region, vaccination substantially reduced the number of measles cases from the 1990s to 2009. However, serious outbreaks of measles caused by genotype D4 MeV unexpectedly occurred across Europe between 2010 and 2012 after the introduction of the D4-Enfield variant or “named strain” in 2008 [1,2]. This variant was named after a strain detected in the UK in 2007 (MVs/Enfield.GBR/14.07/), which spread through Europe, generating other successful “named strains” such as MVs/Hamburg.DEU/03.09/ and MVs/Manchester.GBR/10.09/, amongst others [1,2]. Similarly in Spain, large outbreaks mainly caused by genotype D4 occurred in 2011 and 2012, affecting 4,731 individuals, after several years of low incidence of the disease [3, 4].

MeV is a negative-sense non-segmented RNA virus of the genus *Morbillivirus* from the family *Paramyxoviridae*. The MeV genome has a standard size of 15,894 nucleotides (nt), thus obeying the *rule of six*, that characterizes the morbilliviruses. According to this rule, the total number of nucleotides comprising the MeV genome must be divisible by six for the virus to be viable. The MeV genome contains six transcription units that encode eight proteins (N, P/V/C, M, F, H and L), separated by intergenic regions of 3 nt [5]. Each transcription unit contains a coding region preceded and followed by untranslated regions (UTRs) including the gene start signal and the gene end signal, respectively. The M-F non-coding region (M-F NCR) of the MeV genome comprises a 426-nt 3’ UTR of the matrix protein gene (M 3’UTR), an intergenic region of 3 nt, and a 583-nt 5’ UTR of the fusion protein gene (F 5’UTR). This is the longest non-coding region of the MeV (1012 nt), and it is rich in G-C- with homopolymeric sequences [5, 6]. The functionality of the M-F NCR of the morbilliviruses is not well understood, but it has been suggested that secondary structures are involved in regulating translation or mRNA location [7, 8]. Although the M-F NCR of MeV is not essential for virus replication, it has been associated with cytopathogenicity and the regulation of virus replication by modulating M and F protein expression [9]. A long M 3’UTR promotes M protein translation inducing efficient replication, and a long F 5’UTR discourages F protein translation, reducing the cytopathogenicity of the virus. The M-F NCR region is one of the most variable regions of the MeV genome [6, 10] and has recently been proposed as a new target for MeV molecular characterization [11].

Non-standard length M-F NCR sequences, with a 7-nt insertion in the M 3’ UTR and a deletion of 1 nt in the F 5’UTR, have been identified in D4 genotype MeV strains [12]. These atypical strains were found in cases imported from Europe and India to the USA between 2007 and 2010. A deviation from standard genome organization has recently been noted in 11 MeV genomes obtained from the GenBank database [13], ten of which belong to clade D. Nine of these genomes presented one indel (an insertion or a deletion) in a 28 nt-long homopolymeric region in the F 5’ UTR, and three of them contained the previously described non-standard length M-F NCRs [12].

To investigate the circulation of MeV strains with non-standard length M-F NCR in Spain and to extend our knowledge of the specific genetic features in the M-F NCR in MeV D4 genotype strains, samples from patients infected before and during the major outbreaks of 2011 and 2012 were investigated.

## Materials and methods

### Ethics statement

The samples used in this work were obtained in the context of the National Measles and Rubella Elimination Plan and used in accordance with the requirements of Spanish biomedical research law (Ley 14/2007 de Investigación Biomédica). The protocol was approved by the Comité de Ética de la Investigación y de Bienestar Animal of the Instituto de Salud Carlos III (approval no. CEI PI 35–2015).

### Clinical specimens and epidemiological data

A total of 75 clinical samples identified as genotype D4 collected between 2004 and 2012 [2004 (n = 2), 2005 (n = 1), 2007 (n = 1), 2008 (n = 3), 2010 (n = 1), 2011 (n = 29) and 2012 (n = 38)], belonging to the collection of the National Reference Laboratory for Measles and Rubella at the Centro Nacional de Microbiología of the Instituto de Salud Carlos III (CNM-ISCIII, Spain), were included in the study (Table 1). Epidemiological data related to cases and outbreaks of measles were obtained from the National Network of Epidemiological Surveillance (RENAVE) for years 2008 to 2012.

**Table 1:** Molecular characterization of the samples analyzed in the study.

ID	Sample	N450 Named Strain/ Group of identical N450 sequences ^a^	Group of identical M-F NCR sequences	Indel type^b^	Origin	YEAR
1	MVs/Baleares.ESP/36.04/	MVs/Baleares.ESP/36.04/	MVs/Baleares.ESP/36.04/	Type 8	ThroatSwab	2004
2	MVs/Baleares.ESP/38.04/	MVs/Baleares.ESP/36.04/	MVs/Baleares.ESP/36.04/	Type 8	ThroatSwab	2004
3	MVs/Barcelona.ESP/31.05/	MVs/Barcelona.ESP/31.05/	MVs/Barcelona.ESP/31.05/	Type 8	Urine	2005
4	MVs/Soria.ESP/12.07/	MVs/Soria.ESP/12.07/	MVs/Soria.ESP/12.07/	Type 8	ThroatSwab	2007
5	MVs/Cadiz.ESP/13.08/2	**MVs/Enfield.GBR/14.07/**	MVs/Cadiz.ESP/13.08/2	Type 1	Urine	2008
6	MVs/Cadiz.ESP/14.08/2	MVs/Cadiz.ESP/11.08/	MVs/Cadiz.ESP/13.08/2	Type 1	Urine	2008
7	MVs/Malaga.ESP/36.08/	MVs/Malaga.ESP/36.08/	MVs/Malaga.ESP/36.08/	Type 1	Urine	2008
8	MVs/Madrid.ESP/46.10/	**MVs/Madrid.ESP/46.10/**	MVs/Madrid.ESP/46.10/	Type 2	Urine	2010
9	MVs/Sevilla.ESP/1.11/	**MVs/Madrid.ESP/46.10/**	MVs/Sevilla.ESP/1.11/	Type 2	Urine	2011
10	MVs/Madrid.ESP/11.11/5	**MVs/Manchester.GBR/10.09/**	MVs/Madrid.ESP/11.11/5	Type 2	ThroatSwab	2011
11	MVs/Alicante.ESP/12.11/	**MVs/Manchester.GBR/10.09/**	MVs/Alicante.ESP/12.11/	Type 2	ThroatSwab	2011
12	MVs/Madrid.ESP/13.11/4	**MVs/Manchester.GBR/10.09/**	MVs/Madrid.ESP/11.11/5	Type 2	ThroatSwab	2011
13	MVs/Madrid.ESP/14.11/	**MVs/Manchester.GBR/10.09/**	MVs/Madrid.ESP/46.10/	Type 2	ThroatSwab	2011
14	MVs/Valencia.ESP/16.11/	**MVs/Madrid.ESP/46.10/**	MVs/Valencia.ESP/16.11/	Type 2	Urine	2011
15	MVs/Madrid.ESP/20.11/2	MVs/Madrid.ESP/17.11/	MVs/Madrid.ESP/20.11/2	Type 1	ThroatSwab	2011
16	MVs/Madrid.ESP/21.11/2	**MVs/Manchester.GBR/10.09/**	MVs/Madrid.ESP/21.11/2	Type 5	ThroatSwab	2011
17	MVs/Madrid.ESP/21.11/5	MVs/Enfield.GBR/30.07/	MVs/Madrid.ESP/21.11/5	Type 1	ThroatSwab	2011
18	MVs/Madrid.ESP/21.11/10	**MVs/Manchester.GBR/10.09/**	MVs/Madrid.ESP/21.11/10	Type 5	ThroatSwab	2011
19	MVs/Alicante.ESP/22.11/	**MVs/Manchester.GBR/10.09/**	MVs/Sevilla.ESP/1.11/	Type 2	Urine	2011
20	MVs/Madrid.ESP/23.11/4	**MVs/Manchester.GBR/10.09/**	MVs/Madrid.ESP/46.10/	Type 2	ThroatSwab	2011
21	MVs/Madrid.ESP/24.11/	**MVs/Manchester.GBR/10.09/**	MVs/Madrid.ESP/24.11/	Type 2	ThroatSwab	2011
22	MVs/Madrid.ESP/24.11/5	**MVs/Manchester.GBR/10.09/**	MVs/Madrid.ESP/24.11/5	Type 4	ThroatSwab	2011
23	MVs/Melilla.ESP/25.11/2	**MVs/Enfield.GBR/14.07/**	MVs/Melilla.ESP/25.11/2	Type 1	Urine	2011
24	MVi/Sevilla.ESP/25.11/	**MVs/Madrid.ESP/46.10/**	MVi/Sevilla.ESP/25.11/	Type 2	ThroatSwab	2011
25	MVs/SantaCruzTenerife.ESP/27.11/	**MVs/Madrid.ESP/46.10/**	MVs/SantaCruzTenerife.ESP/27.11/	Type 2	ThroatSwab	2011
26	MVs/Vizcaya.ESP/29.11/	**MVs/Manchester.GBR/10.09/**	MVs/Vizcaya.ESP/29.11/	Type 4	ThroatSwab	2011
27	MVs/Valencia.ESP/31.11/	**MVs/Madrid.ESP/46.10/**	MVs/Sevilla.ESP/1.11/	Type 2	Urine	2011
28	MVs/Valencia.ESP/31.11/2	**MVs/Madrid.ESP/46.10/**	MVs/Sevilla.ESP/1.11/	Type 2	Urine	2011
29	MVs/Madrid.ESP/34.11/	MVs/Enfield.GBR/30.07/	MVs/Madrid.ESP/34.11/	Type 1	ThroatSwab	2011
30	MVs/Madrid.ESP/34.11/2	MVs/Enfield.GBR/30.07/	MVs/Madrid.ESP/21.11/5	Type 1	ThroatSwab	2011
31	MVs/Madrid.ESP/37.11/	MVs/Enfield.GBR/30.07/	MVs/Madrid.ESP/37.11/	Type 1	ThroatSwab	2011
32	MVs/Valencia.ESP/41.11/	**MVs/Madrid.ESP/46.10/**	MVs/Sevilla.ESP/1.11/	Type 2	Urine	2011
33	MVs/Madrid.ESP/45.11/8	MVs/Enfield.GBR/30.07/	MVs/Madrid.ESP/21.11/5	Type 1	ThroatSwab	2011
34	MVs/Valencia.ESP/45.11/	**MVs/Madrid.ESP/46.10/**	MVs/Valencia.ESP/45.11/	Type 4	Urine	2011
35	MVs/Valencia.ESP/45.11/3	**MVs/Madrid.ESP/46.10/**	MVs/Sevilla.ESP/1.11/	Type 2	Urine	2011
36	MVs/Valencia.ESP/47.11/	**MVs/Madrid.ESP/46.10/**	MVs/Sevilla.ESP/1.11/	Type 2	Urine	2011
37	MVs/Valencia.ESP/47.11/3	**MVs/Madrid.ESP/46.10/**	MVs/Sevilla.ESP/1.11/	Type 2	Urine	2011
38	MVs/LasPalmas.ESP/2.12/	MVs/Enfield.GBR/30.07/	MVs/Madrid.ESP/21.11/5	Type 1	ThroatSwab	2012
39	MVs/Madrid.ESP/4.12/	MVs/Madrid.ESP/4.12/	MVs/Madrid.ESP/4.12/	Type 3	ThroatSwab	2012
40	MVs/Alicante.ESP/4.12/3	**MVs/Madrid.ESP/46.10/**	MVs/Sevilla.ESP/1.11/	Type 2	Urine	2012
41	MVs/Alicante.ESP/06.12/	**MVs/Madrid.ESP/46.10/**	MVs/Sevilla.ESP/1.11/	Type 2	ThroatSwab	2012
42	MVs/Alicante.ESP/6.12/5	**MVs/Madrid.ESP/46.10/**	MVs/Sevilla.ESP/1.11/	Type 2	Urine	2012
43	MVs/Baleares.ESP/06.12/	**MVs/Madrid.ESP/46.10/**	MVs/Sevilla.ESP/1.11/	Type 2	ThroatSwab	2012
44	MVs/Alicante.ESP/6.12/9	MVs/Alicante.ESP/5.12/	MVs/Sevilla.ESP/1.11/	Type 2	Urine	2012
45	MVs/Alicante.ESP/7.12/6	MVs/Alicante.ESP/5.12/	MVs/Sevilla.ESP/1.11/	Type 2	Urine	2012
46	MVs/Alicante.ESP/7.12/7	MVs/Alicante.ESP/5.12/	MVs/Sevilla.ESP/1.11/	Type 2	Urine	2012
47	MVs/Madrid.ESP/7.12/11	**MVs/Madrid.ESP/46.10/**	MVs/Sevilla.ESP/1.11/	Type 2	ThroatSwab	2012
48	MVs/Madrid.ESP/8.12/4	**MVs/Madrid.ESP/46.10/**	MVs/Sevilla.ESP/1.11/	Type 2	ThroatSwab	2012
49	MVs/Baleares.ESP/9.12	**MVs/Madrid.ESP/46.10/**	MVs/Sevilla.ESP/1.11/	Type 2	ThroatSwab	2012
50	MVs/Baleares.ESP/10.12/	**MVs/Madrid.ESP/46.10/**	MVs/Sevilla.ESP/1.11/	Type 2	Urine	2012
51	MVs/Alicante.ESP/10.12/	**MVs/Madrid.ESP/46.10/**	MVs/Sevilla.ESP/1.11/	Type 2	Placenta	2012
52	MVs/Baleares.ESP/11.12/	**MVs/Madrid.ESP/46.10/**	MVs/Sevilla.ESP/1.11/	Type 2	ThroatSwab	2012
53	MVs/Baleares.ESP/13.12/2	**MVs/Madrid.ESP/46.10/**	MVs/Sevilla.ESP/1.11/	Type 2	ThroatSwab	2012
54	MVs/Navarra.ESP/12.12/	**MVs/Marmande.FRA/43.11/2**	MVs/Navarra.ESP/12.12/	Type 6	ThroatSwab	2012
55	MVs/Baleares.ESP/13.12/5	MVs/Baleares.ESP/13.12/5	MVs/Sevilla.ESP/1.11/	Type 2	ThroatSwab	2012
56	MVs/Baleares.ESP/14.12/4	**MVs/Madrid.ESP/46.10/**	MVs/Sevilla.ESP/1.11/	Type 2	ThroatSwab	2012
57	MVs/Baleares.ESP/14.12/	MVs/Baleares.ESP/14.12/	MVs/Sevilla.ESP/1.11/	Type 2	ThroatSwab	2012
58	MVs/Baleares.ESP/14.12/2	MVs/Baleares.ESP/14.12/2	MVs/Baleares.ESP/14.12/2	Type 2	ThroatSwab	2012
59	MVs/Baleares.ESP/14.12/3	**MVs/Madrid.ESP/46.10/**	MVs/Sevilla.ESP/1.11/	Type 2	ThroatSwab	2012
60	MVs/Baleares.ESP/14.12/5	MVs/Baleares.ESP/14.12/2	MVs/Sevilla.ESP/1.11/	Type 2	ThroatSwab	2012
61	MVs/Baleares.ESP/15.12/4	**MVs/Madrid.ESP/46.10/**	MVs/Sevilla.ESP/1.11/	Type 2	ThroatSwab	2012
62	MVs/Navarra.ESP/15.12/2	**MVs/Marmande.FRA/43.11/2**	MVs/Navarra.ESP/15.12/2	Type 6	ThroatSwab	2012
63	MVs/Baleares.ESP/16.12/	**MVs/Madrid.ESP/46.10/**	MVs/Sevilla.ESP/1.11/	Type 2	ThroatSwab	2012
64	MVs/Baleares.ESP/17.12/	MVs/Baleares.ESP/14.12/2	MVs/Sevilla.ESP/1.11/	Type 2	ThroatSwab	2012
65	MVs/Baleares.ESP/17.12/2	**MVs/Madrid.ESP/46.10/**	MVs/Baleares.ESP/17.12/2	Type 2	ThroatSwab	2012
66	MVs/Baleares.ESP/17.12/3	**MVs/Madrid.ESP/46.10/**	MVs/Sevilla.ESP/1.11/	Type 2	ThroatSwab	2012
67	MVs/Baleares.ESP/19.12/	**MVs/Madrid.ESP/46.10/**	MVs/Sevilla.ESP/1.11/	Type 2	ThroatSwab	2012
68	MVs/Baleares.ESP/20.12/2	**MVs/Madrid.ESP/46.10/**	MVs/Sevilla.ESP/1.11/	Type 2	ThroatSwab	2012
69	MVs/Baleares.ESP/21.12/	**MVs/Madrid.ESP/46.10/**	MVs/Baleares.ESP/21.12/	Type 5	ThroatSwab	2012
70	MVs/Baleares.ESP/22.12/	**MVs/Madrid.ESP/46.10/**	MVs/Sevilla.ESP/1.11/	Type 2	ThroatSwab	2012
71	MVs/Baleares.ESP/22.12/4	MVs/Baleares.ESP/22.12/4	MVs/Sevilla.ESP/1.11/	Type 2	ThroatSwab	2012
72	MVs/Baleares.ESP/23.12/2	**MVs/Madrid.ESP/46.10/**	MVs/Sevilla.ESP/1.11/	Type 2	ThroatSwab	2012
73	MVs/Baleares.ESP/23.12/	**MVs/Madrid.ESP/46.10/**	MVs/Baleares.ESP/23.12/	Type 5	ThroatSwab	2012
74	MVs/Baleares.ESP/25.12/	**MVs/Madrid.ESP/46.10/**	MVs/Baleares.ESP/25.12/	Type 2	ThroatSwab	2012
75	MVs/Baleares.ESP/25.12/2	**MVs/Madrid.ESP/46.10/**	MVs/Baleares.ESP/25.12/2	Type 7	ThroatSwab	2012

^a^Named strains available in MeaNS are shown in bold.

^b^Types according to the characteristics of the indel region.

### Amplification and sequencing of targets

The highly variable 450-nt fragment coding for the carboxyl terminus of the nucleocapsid protein (N-450), which has been defined by the WHO for genotyping, was amplified as previously described [14]. The sequences obtained were compared to the MeaNS database [15] to identify any specific MeV variant, recently defined as “named strains” according to their geographic and temporal dissemination [16]. Each set of identical sequences that was not linked to any “named strain” described in MeaNS were named with the earliest sequence name. The M-F NCR region was amplified and sequenced. Four primers were designed, based upon the consensus sequence obtained from all complete MeV genomes in GenBank: MV_F1 (5’-CAAGATAGTAAGAATCCAGGCAG) and MV_F2 (5’ -CGTGATCATAAATGATGACCAAGGAC) as forward primers and MV_R1 (5’- ACTTTGTAGCTTGCACTTCC) and MV_R2 (5’-TTGTAGCTTGCACTTCCTAYYCC) as reverse primers. RT-PCR was performed using the OneStep RT-PCR kit (QIAGEN) according to the manufacturer’s instructions, with 0.6 μM of each primer and 400 μM of dNTPs, and including buffer Q as adjuvant. The amplifying conditions were 50°C for 30 min, followed by 95°C for 15 min and 40 cycles of 94°C for 30 s, 55°C for 60 s and 72°C for 90 s, finishing at 72°C for 10 min. The nested PCR was performed using the BioTAQ DNA polymerase (Bioline, London, UK) according to the manufacturer’s recommendations, with 0.4 μM of each primer, 200 μM of dNTPs and 2 mM of Cl_2_Mg, using 1M of betaine (Sigma-Aldrich, St. Louis, MO, USA) as adjuvant. The amplification conditions included an initial denaturation step at 94°C for 2 min, followed by 30 cycles of 94°C for 30 s, 58°C for 30 s and 72°C for 80 s, and a final extension performed at 72°C for 7 min. Amplicons were purified using Illustra ExoProStar 1-Step (GE Health Care Life Science, Freiburg, Germany) according to the manufacturer’s instructions. Amplicons were sequenced with the ABI Big Dye Terminator Cycle Sequencing Kit (Applied Biosystems, Branchburg, NJ, USA) using the MV_F2 and MV_R2 primers described above, and the additional primers MV_F4 (5’-AAACTTAGGGCCAAGGAAYAYAC) and MV_R4 (5’-TTGCCGTGGTSKTGTG), which were designed to cover the central part of the M-F NCR sequence, and MV_Fsec_D (5’-GACCCAGACCACCAACC), which was designed to confirm the homopolymeric sequence in the M 3’ UTR.

### Analysis of the M-F NCR

To analyze the M-F NCR, the existing complete MeV genomes (n = 118) and MeV M-F NCR sequences (n = 53) were obtained from GenBank (accessed in January 2017). Eight MeV genomes and 19 MeV M-F NCRs belonged to the D4 genotype. All these deposited sequences and those obtained in this study were aligned using MAFTT v.7 [17] and edited using BioEdit v.7.2.5 [18] to extract the M-F NCRs for subsequent analysis. Each set of identical sequences was identified using DNAsp v5 software [19].

### Phylogenetic analysis

The sequences obtained in this study were aligned with MAFTT v.7, including genotype D4 and D8 reference sequences from GenBank. D8 genotype sequences were used as outgroup. Phylogenetic trees were built using the maximum likelihood method with MEGA v.6 [20]. The Kimura 2P for the N-450 target and Tamura-Nei with gamma distribution for the M-F NCR target were the most suitable evolutionary models identified by MEGA v.6 and so were chosen for use in the analysis. The reliability of the phylogenetic analysis at each branch node was estimated by the bootstrap method using 1000 replications.

### GenBank accession numbers

The sequences obtained in this study have been deposited in GenBank with the accession numbers KX518607-KX518619 and KX499400 for N-450, and KX525239-KX525321 for M-F NCR.

## Results

**Fig 1:**
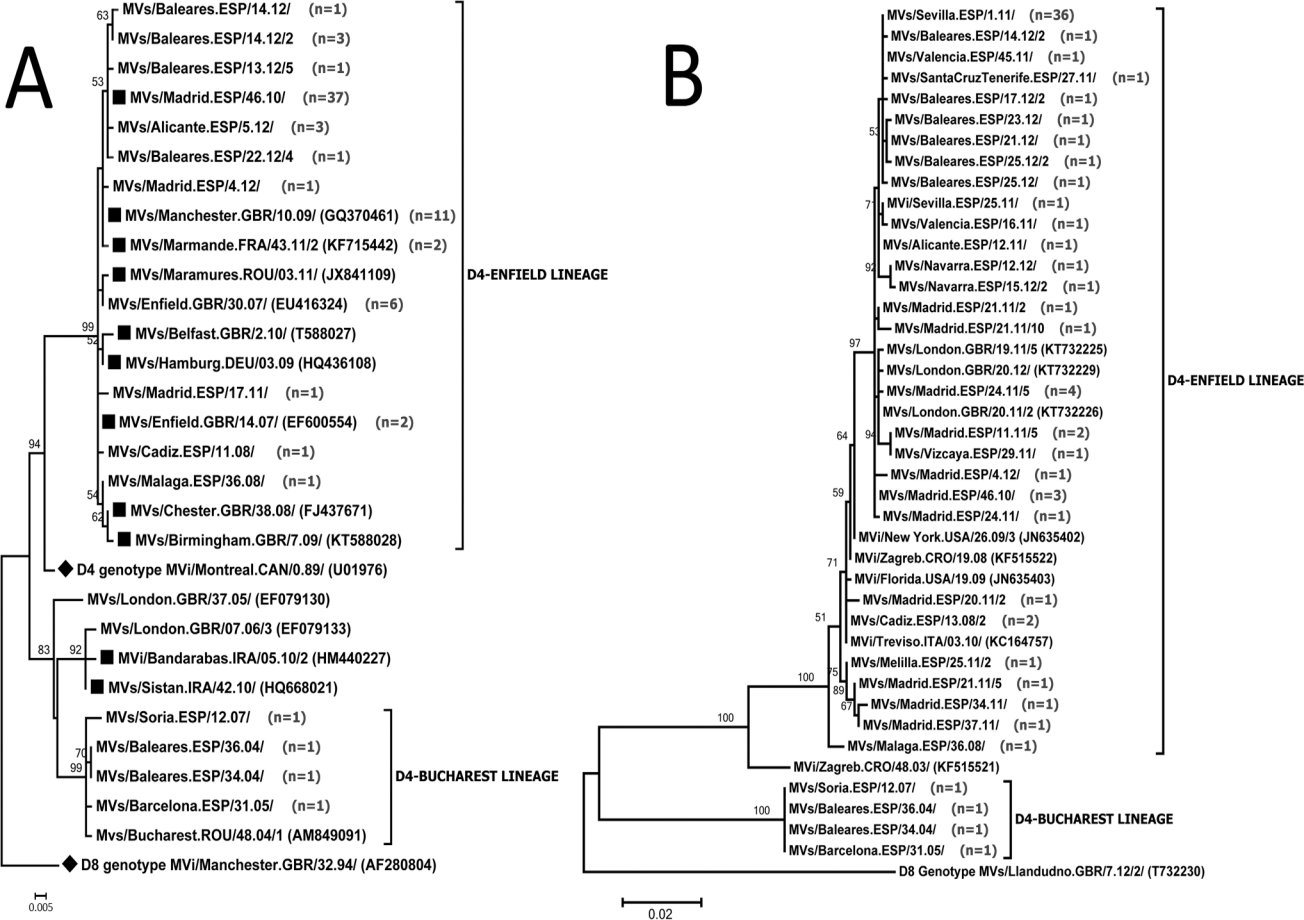
Phylogenetic analysis of the N-450 and M-F NCR. Panel A: N-450 dendrogram. The Kimura 2P nucleotide substitution model was used to build the tree. The MeV genotype D8 and D4 references (black diamonds) and D4 named strains accepted in MeaNS (black squares) were included in the analysis. The tree was rooted with respect to the genotype D8 WHO reference sequence. The number (n) of sequences of each set of identical sequences or named strain is shown in brackets. Panel B: M-F NCR dendrogram. The TN93 nucleotide substitution model with gamma distribution was used to build the tree. Genotype D4 M-F NCR sequences from GenBank were included in the analysis. A genotype D8 M-F NCR sequence from GenBank was used to root the tree. The number (n) of identical sequences of each set of identical sequences or variant is shown.

Analysis of the N-450 sequences identified 17 different sets of identical sequences, all of which belonged to the D4 genotype. Three of these had already been defined as named strains in the MeaNS database and belonged to the same genetic lineage, according to the topology of the phylogenetic tree (Fig 1, Panel A), named here as D4-Enfield lineage: MVs/Enfield.GBR/14.07/, MVs/Manchester.GBR/10.09/, and Marmande.FRA/43.11/2. The most frequent set of identical sequences (37 samples) was the MVs/Madrid.ESP/46.10/ (Table 2). This set of identical sequences belonging to the D4-Enfield lineage was circulating in Spain from 2010 to 2012 and was accepted as a named strain in MeaNS database. The MVs/Madrid.ESP/46.10/ has two characteristic mutations on the N-450 sequence compared to the MVs/Enfield.GBR/14.07/ N-450 sequence (C260T and, T441A), sharing one of them with the N-450 sequence of the MVs/Manchester.GBR/10.09/ named strain (C260T). Five sets of identical N-450 sequences cluster to a phylogenetical clade with the MVs/Madrid.ESP/46.10/ named strain (Fig 1, Panel A) and presented the two characteristic mutations. The other set of identical sequences of years 2008 to 2012 identified in this study also clustered within the D4-Enfield lineage (Fig 1, Panel A). The four samples collected before 2008 clustered in the previously described D4-Bucharest lineage [1].

**Table 2:** Number of sequences on each set of identical N450 and M-F NCR sequences associated to the MVs/Madrid.ESP/46.10/ sub-lineage.

	Set of identical N-450*						
Set of identical M-F NCR*	*MVs/Madrid*.*ESP/46*.*10/*	*MVs/Alicante*. *ESP/5*.*12/*	*MVs/Baleares*.*ESP/13*.*12/5*	*MVs/Baleares*.*ESP/14*.*12/*	*MVs/Baleares*. *ESP/14*.*12/2*	*MVs/Baleares*. *ESP/22*.*12/4*	*Total*
***MVs/Madrid*.*ESP/46*.*10/***	*1*						*1*
***MVs/Sevilla*.*ESP/1*.*11/***	***27***	*3*	*1*	*1*	*2*	*1*	*35*
***MVs/Valencia*.*ESP/16*.*11/***	*1*						*1*
***MVi/Sevilla*.*ESP/25*.*11/***	*1*						*1*
*MVs/SantaCruzTenerife*.*ESP/27*.*11/*	*1*						*1*
***MVs/Valencia*.*ESP/45*.*11/***	*1*						*1*
***MVs/Baleares*.*ESP/14*.*12/2***					*1*		*1*
***MVs/Baleares*.*ESP/17*.*12/2***	*1*						*1*
***MVs/Baleares*.*ESP/21*.*12/***	*1*						*1*
***MVs/Baleares*.*ESP/23*.*12/***	*1*						*1*
***MVs/Baleares*.*ESP/25*.*12/***	*1*						*1*
***MVs/Baleares*.*ESP/25*.*12/2***	*1*						*1*
*Total number of sequences*	*37*	*3*	*1*	*1*	*3*	*1*	*46*

*Sets of identical sequences belonged to epidemiologically linked outbreaks are shown in bold.

Analysis of the M-F NCR target identified 30 different sets of identical sequences (Fig 1, Panel B). Twelve of them were found in samples with the MVs/Madrid.ESP/46.10/ variant or associated sets of identical N-450 sequences (Table 2, Fig 1, Panel A). All cases except one belonged to epidemiologically linked outbreaks of MVs/Madrid.ESP/46.10/ sub-lineage [3, 4]. The associated sets of identical sequences arose in the context of the outbreaks. All of them displayed either the MF NCR sequence MVs/Sevilla.ESP/1.11/ or associated sequences (Table 2).

**Fig 2:**
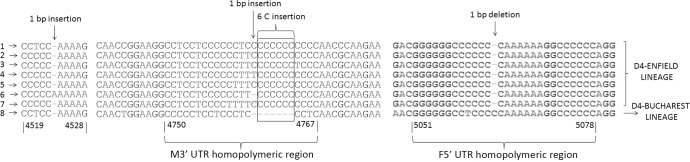
M-F NCR types. Eight types of nucleotide sequences were identified in this study. Type 8 corresponds to the sequences of the D4-Bucharest lineage, which did not show the atypical M-F NCR structure. Numbers under the alignment indicate nucleotide positions in the full-length genome of the MeV Edmonston strain (GenBank Accession No. AY486083).

Sequences from patients obtained before 2008 clustered in a different clade from the more recent ones (Fig 1, Panel B), at a similar location to that observed in the N-450 dendrogram. Interestingly, indels similar to those described in MeV strains with non-standard length M-F NCR imported to the USA from Europe and India [12] were observed in all the studied samples (Fig 2, type 1–7), except those from the four patients infected before 2008 (Fig 2, type 8), who had a standard length M-F NCR.

All Spanish samples analyzed in this study containing non-standard length M-F NCR featured a 1-nt deletion in the F 5’UTR (Fig 2), which was located on the recently described 28 nt-long homopolymeric region [positions 5051–5078 referenced to the Edmonston strain (GenBank Accession No. AY486083)] [13]. Moreover, there was an insertion of 6 cytosines (C) in the M 3’ UTR located in a C-rich homopolymeric region of 18 nt (positions 4750–4767 referenced to the Edmonston strain; GenBank Accession No. AY486083) that were flanked by two conserved guanidines (G) and two conserved adenines (A) at the 5’ and 3’ locations, respectively (Fig 2). A C insertion was also identified in the same homopolymeric region (position 4764), and there was a T insertion in one type (Fig 2, type 4). These insertions ensured that the *rule of six* is obeyed [5]. In the case of the M-F NCR sequences of the MeV strains belonging to the N-450 named strain MVs/Marmande.FRA/43.11/2, this insertion was located in the M 3’ UTR outside the homopolymeric region, with an A at position 4524 (Fig 2, type 6). This M 3’ UTR homopolymeric region of the MeV strains with non-standard length M-F NCR was quite variable amongst the various sets of identical M-F NCR sequences identified in this study, showing up to 7 different types of nucleotide sequences (Fig 2).

**Fig 3:**
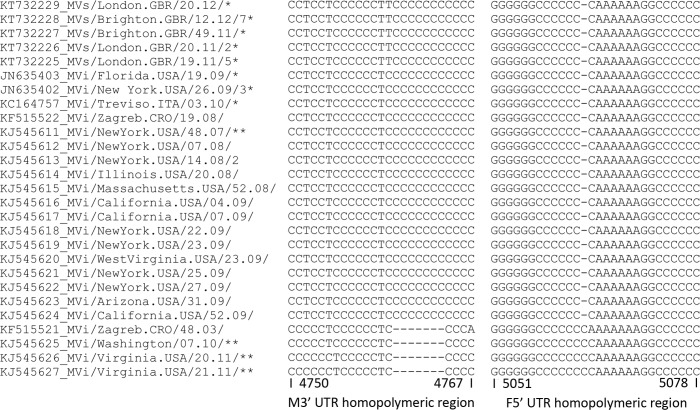
M-F NCR in the MeV D4 genotype strains obtained from GenBank. 27 M-F NCRs of MeV genotype D4 strains were identified in GenBank; 23 had non-standard length M-F NCR sequences, 22 were of European origin, and 1 was from India. * indicates that the M-F NCR was extracted from the complete genome sequence. ** indicates MeV cases imported from India.

In addition, 27 M-F NCRs of MeV genotype D4 strains were identified in GenBank; 23 had non-standard length M-F NCR sequences, including one isolate from Italy, one from Croatia, five from UK, and 16 isolates from the USA (Fig 3), of which 15 were cases imported from Europe and one was from India [12]. All non-standard length M-F NCRs obtained from GenBank belonged to type 1, with the exception of the five sequences from the UK, which belonged to type 2. The remaining four strains had a standard length M-F NCR sequence, one of them having been isolated in Europe in 2003 (MVi/Zagreb.CRO/48.03/) and three in USA, although these were cases imported from India (Fig 3).

## Discussion

After several years with a low incidence of measles cases, large outbreaks occurred in Europe between 2010 and 2012 after the introduction of the D4-Enfield lineage at the end of 2007, which replaced the previously circulating D4-Bucharest lineage viruses [1,2]. We have also observed this replacement in Spain, whereby all viruses from samples collected after 2008 belonged to the D4-Enfield lineage, whilst the older ones were of the D4-Bucharest lineage.

The reasons for the successful spread of the D4-Enfield lineage MeV in Western Europe [2] are not well understood. The development of major measles outbreaks is related to the presence of susceptible population groups in which the virus can spread easily. However, vaccination coverage in Western Europe and Spain was already high before 2010–2012, when these large outbreaks occurred [3,4]. Among the factors that might have contributed to this widespread MeV dissemination could be the special features of the viruses themselves. Recently, MeV strains with non-standard length M-F NCR sequences, belonging to genotype D4, were discovered in USA in cases imported from Europe and India [12]. Similarly, we have identified non-standard length M-F NCR sequences in all the samples analyzed containing MeV from the D4-Enfield lineage, including four named strains. Since the characterization had been done directly from clinical samples, these atypical M-F NCRs could not have been the consequence of selection bias during virus isolation.

All of the non-standard length M-F NCR sequences identified in this study had a net gain of 6 nt, thus obeying the *rule of six* [5]. All of them have a 1-nt deletion located at the recently described homopolymeric region of the F 5’ UTR [13]. In addition, the non-standard length M-F NCR sequences presented an insertion of 6C in a C-rich homopolymeric region at the M 3’ UTR and a 1-nt insertion in the 5’ upstream fragment of the same homopolymeric region, resulting in the previously described 7-nt insertion [12]. The exceptions were the sequences belonging to the N-450 named strain MVs/Marmande.FRA/43.11/2, whose 1-nt insertion lies outside the M homopolymeric region in order to fulfill the *rule of six*. Other MeVs of this latter named strain should be analyzed to establish whether they have the same genetic structure in the M-F NCR.

These genetic features were not present in the MeVs belonging to the D4-Bucharest lineage that circulated previously in Spain. We obtained information only from four samples. Unfortunately, attempts to amplify the M-F NCR from more clinical samples obtained before 2008 were unsuccessful, probably due to the long time the available samples had been in storage.

The analysis of the M-F NCR of MeV D4 strains obtained from GenBank produced similar results. All the MeV D4 strains of European origin deposited in GenBank since the end of 2007 presented these atypical M-F NCR sequences. In addition, only one M-F NCR from a European MeV D4 strain obtained before the expansion of the D4-Enfield lineage was found in GenBank (MVi/Zagreb.CRO/48.03), and this did not contain any atypical indels. These findings suggest that non-standard length M-F NCR sequences is a specific genetic feature associated with members of the D4-Enfield lineage.

The origin of the D4-Enfield lineage is unclear. It was named after a strain detected in the UK in 2007 (MVs/Enfield.GBR/14.07). However, the oldest known sequence of the MeV D4-Enfield lineage is from India (MVs/Raichur.IND/38.06/, No. EU812270), suggesting that this lineage was present there. This explains how a MeV D4 genotype strain with non-standard length M-F NCR was imported from India into the USA in 2009 [12].

The effect of these atypical genomes on the pathogenesis of MeV is unknown, although the M-F NCR has been implicated in the regulation of the viral replication and cytopathogenicity [9]. The functional significance of the 6C insertions needs further exploration, but a longer M 3’UTR would promote virus replication by increasing M protein expression [9]. To date, the biological characteristics have only been examined in two non-standard genome length isolates, in two different cell lines showing no differences in plaque size or replication efficiency [12]. However, further functional studies using several viral isolates from different named strains of the D4-Enfield lineage and showing different types of homopolymeric regions at the M 3’ UTR are needed to evaluate the biological significance of this these atypical M-F NCR sequences. In addition, whole-genome sequencing of these MeV isolates may provide information about other specific genetic features of these atypical strains.

Recently, the use of new sequencing windows, including the M-F NCR, has been recommended for improving measles surveillance [13, 16]. The results of the phylogenetic analysis of Spanish M-F NCRs suggest higher resolution in discriminating strains than did the N-450 analysis, since more sets of identical sequences were identified with the first. In addition the results of the phylogenetic analysis of the MF-NCR sequences belonging to the named strain MVs/Madrid.ESP/46.10/ and associated sets of identical sequences are consistent with the relationships among the different outbreaks established according to the epidemiological data. It agrees with that previously described in the measles outbreak investigation in Sweden (2013–2014) using the phylogenetic analysis of the M-F NCR [11]. These results suggest a potential use of this region as a complementary tool to epidemiological investigation. According to the higher variability of this region, it could provide better discrimination of chains of transmission than other regions of the MeV genome. Further studies should be made on cases with the same N-450 sequence without an epidemiological link, such as those detected in the context of outbreaks with different geographical and temporal origin.

The analysis of the M-F NCR region for exploring MeV transmission and for the surveillance of potentially emerging strains with non-standard length M-F NCR is strongly recommended as part of future strategies for measles elimination.
